# Blast Exposure Dysregulates Nighttime Melatonin Synthesis and Signaling in the Pineal Gland: A Potential Mechanism of Blast-Induced Sleep Disruptions

**DOI:** 10.3390/brainsci12101340

**Published:** 2022-10-04

**Authors:** Manoj Govindarajulu, Mital Y. Patel, Donna M. Wilder, Joseph B. Long, Peethambaran Arun

**Affiliations:** Blast-Induced Neurotrauma Branch, Center for Military Psychiatry and Neurosciences, Walter Reed Army Institute of Research, Silver Spring, MD 20910, USA

**Keywords:** blast injury, circadian rhythm, melatonin, pineal gland, gene expression

## Abstract

Blast-induced traumatic brain injury (bTBI) frequently results in sleep-wake disturbances. However, limited studies have investigated the molecular signaling mechanisms underlying these sleep disturbances, and potentially efficacious therapies are lacking. We investigated the levels of melatonin and genes involved in melatonin synthesis pathway in the pineal glands of Sprague Dawley rats exposed to single and tightly coupled repeated blasts during the night and daytime. Rats were exposed to single and tightly coupled repeated blasts using an advanced blast simulator. The plasma, cerebrospinal fluid (CSF), and pineal gland were collected at 6 h, 24 h, or 1 month postblast at two different time points: one during the day (1000 h) and one at night (2200 h). Differential expressions of genes involved in pineal melatonin synthesis were quantified using quantitative real-time polymerase chain reaction (qRT-PCR). Plasma and CSF melatonin levels were assessed using a commercial melatonin ELISA kit. The plasma and CSF melatonin levels showed statistically significant decreases at 6 h and 24 h in the blast-exposed rats euthanized in the night (in dim light), with no significant alterations noted in rats euthanized in the morning (daylight) at all three-time points. Blast-exposed rats showed statistically significant decreases in Tph1, Aanat, Asmt, and Mtnr1b mRNA levels, along with increased Tph2 mRNA, in the pineal gland samples collected at night at 6 h and 24 h. No significant changes in the mRNA levels of these genes were noted at 1 month. These findings imply that the melatonin circadian rhythm is disrupted following blast exposure, which may be a factor in the sleep disturbances that blast victims frequently experience.

## 1. Introduction

Sleep disturbances are prevalent with subacute and chronic mild traumatic brain injury (TBI), and the sleep disruption can impede recovery from the TBI [1]. Patients with TBI develop sleep–wake disturbances by 6 months after the brain injury, illustrating the high incidence and persistence of effects on sleep [2]. Although sleep disruptions in TBI may be caused by injury to brain regions involved in sleep–wake cycle, the precise mechanisms remain unclear.

Circadian rhythms are 24 h cyclical changes that regulate important biological functions and processes. The sleep–wake cycle is one of the major and well-known examples of circadian rhythms [3]. A disruption in the circadian rhythm due to several reasons, including TBI, can create significant sleep problems such as circadian rhythm sleep disorders, insomnia, and sleep apnea [4,5]. Melatonin synthesis in the pineal gland predominantly occurs on exposure to darkness and is inhibited by light, suggesting that melatonin is involved in circadian rhythmicity and the regulation of the daily sleep–wake cycle [6,7].

Circadian melatonin secretion from the pineal gland is regulated by the suprachiasmatic nucleus (SCN) of the hypothalamus through a series of positive and negative feedback loops, leading to the expression of several genes (core clock genes). Melatonin acts on the SCN and attenuates the wake-promoting signal of the circadian clock, thereby promoting sleep [8]. The positive loop is composed of the Brain and Muscle ARNT-like 1 (BMAL1) gene and the Circadian Locomotor Output Cycles Kaput (CLOCK) gene, which activate the transcription of the repressor genes Period (Per) and Cryptochrome (Cry). The PER and CRY proteins translocate back into the nucleus and inhibit their own expression by suppressing the transactivation of CLOCK/BMAL1, thereby closing the feedback loop (negative loop) [9,10]. As PER and CRY proteins are gradually degraded, the repression of BMAL1 and CLOCK is relieved. One entire cycle of this autofeedback loop lasts 24 h and hence regulates the circadian rhythm [11]. Apart from maintaining the sleep–wake cycle, the circadian rhythm modulates several physiological functions such as body temperature, energy regulation, and hormone secretion. The hypothalamic–pituitary–adrenal (HPA) axis and the hypothalamic–pituitary–gonadal (HPG) axis are the major neuroendocrine systems that regulate circadian hormonal release [12]. These physiological functions are driven by clock genes, which can affect pulsatile sexual hormone release, thereby regulating fertility and reproduction [13,14].

Melatonin synthesis involves the conversion of the amino acid tryptophan through the intermediate metabolite serotonin and is initiated by the enzyme tryptophan hydroxylase, which is essential for normal sleep/wake cycles. Aralkylamine-N-acetyltransferase (AANAT) and acetylserotonin O-methyltransferase (ASMT) are responsible for the conversion of serotonin to melatonin [15]. The activity of AANAT increases up to 100-fold in rat pineal glands during the night after turning off the lights, and its activity decreases sharply to the daytime levels within a few minutes after light is re-established [16,17]. The increase in pineal AANAT activity is directly proportional to the levels of melatonin in the pineal gland and in the blood circulation [18]. Furthermore, in addition to AANAT, the enzymatic activity of ASMT has been shown to regulate the level of melatonin synthesis in the pineal gland at night [19]. Taken together, these observations point to the important role of AANAT and ASMT in the maintenance of the circadian melatonin rhythm, which affects normal sleep–wake cycles.

The use of improvised explosive devices and other explosives in recent military conflicts has led to a marked increase in blast-related TBI (bTBI) in warfighters and in civilians [20,21,22]. Exposure to a blast creates a wide spectrum of injuries, ranging from mild effects to life threatening injuries. Sleep disturbances can develop acutely following injury or emerge later during recovery, at times persisting for years after the initial injury. Previous studies in individuals with TBI have demonstrated decreased nocturnal melatonin synthesis in comparison to healthy individuals, suggesting that brain injury might disrupt the melatonin synthesis pathway, thereby resulting in altered sleep–wake cycles [23]. Furthermore, studies indicate that melatonin levels after TBI remain chronically depressed, and a recent study showed a 42% decrease in overnight melatonin production in individuals with non-blast-related TBI compared to healthy controls [24,25]. Apart from regulating the circadian rhythm, melatonin possesses strong anti-inflammatory, antioxidative, and antiapoptotic properties and can modulate sympathetic and parasympathetic activities. The disruption of the melatonin synthesis pathway by TBI can also cause the dysfunction of extracerebral organs such as the heart. Furthermore, TBI-related cardiac dysfunction can worsen the brain damage and delay the functional recovery [26]. Nevertheless, no prior studies have investigated the effect of bTBI on genes involved in the melatonin synthesis pathway in the pineal gland. The molecular signaling mechanisms involved in sleep and circadian disturbances represent a unique, novel, and modifiable treatment target that can potentially improve outcomes in patients exposed to blasts. Although numerous mechanisms may contribute to sleep disturbances in TBI, studies indicate that in TBI patients reduced evening and overnight melatonin production may indicate a disruption in the circadian regulation of melatonin synthesis [23]. As endogenous melatonin is involved in the circadian control of the sleep–wake cycle, attenuated melatonin profiles may contribute to sleep-related disturbances following TBI, including bTBI. Hence, in this study, we assessed changes in plasma and CSF melatonin levels and the associated transcriptional changes in the genes involved in pineal gland melatonin synthesis at different time points in rats exposed to blast injury.

## 2. Materials and Methods

### 2.1. Animals

Male 7–8-week-old Sprague Dawley rats (weighing 250–275 g) were obtained from Charles River Laboratories. The rats were housed in individually ventilated cages in a dedicated rodent room maintained at 20–22 °C on a 12 h light/12 h dark cycle with free access to standard rat chow diet (Prolab IsoPro RMH3000 from LabDiet, St. Louis, MO, USA) and chlorinated water ad libitum. The lights were turned off at 1800 h and turned back on at 0600 h. All rat experiments were performed at the AAALAC-accredited Walter Reed Army Institute of Research, Silver Spring, MD, under an institutional animal care and use committee (IACUC)-approved protocol. The rats were randomly placed into three groups: sham, single blast (B), and repeated blast (BB), with each group containing 6–8 rats. 

### 2.2. Blast Exposure Details

The rats subjected to blast exposure were preanesthetized with isoflurane (4%) for 6–8 min. The anesthetized rats were secured in a longitudinal prone orientation (rats facing the oncoming blast wave) in the Advanced Blast Simulator (ABS). To induce moderate bTBI, a peak positive static pressure of ~19 psi with a positive phase duration of 4–5 ms was utilized. The rats subjected to tightly coupled repeated blast exposures were exposed to two blast overpressure waves (~19 psi) separated by 2 min, as described in our previous study [27]. 

### 2.3. Blood Sampling, CSF Collection, and Measurement of Melatonin

The rats were anesthetized with isoflurane and euthanized at three time points: 6 h, 24 h, or 1 month after blast injury. Blood samples were collected in BD vacutainer EDTA tubes purchased from Becton, Dickinson and Company, Franklin Lakes, NJ. Plasma was separated by centrifugation at 1000× *g* for 15 min, aliquoted, and stored at −80 °C until analysis. The CSF samples were collected from the cisterna magna, aliquoted, and stored at −80 °C until analysis. The pineal glands were dissected and stored at −80 °C until further analysis. For dark-phase sampling, the samples were collected at 2200 h, and for light-phase sampling, the samples were collected during daytime at 1000 h. Quantitative measurements of melatonin in the plasma and CSF were performed using an Eagle Biosciences direct melatonin ELISA kit (catalogue # MEL31-K01, Amherst, NH, USA) as per the manufacturer’s protocol.

### 2.4. RNA Extraction and Quantitative RT-PCR

High-quality total RNA was extracted from the rat pineal gland using the Qiagen RNeasy mini kit (Cat. No: 74104) and was quantified using a NanoDrop 2000 (Thermo Scientific, Waltham, MA, USA). The RNA samples with absorbance ratios (260/280 nm) of 1.8–2.0 were considered pure and were transcribed into CDNA using an RT2 First Strand Kit (Cat # 330404, Qiagen, Germantown, MD, USA). Until usage, the cDNA samples were stored at −20 °C. All primers and reagents were obtained from QIAGEN. The primers used in the study are outlined in Table 1. All samples were run in triplicate. Briefly, qRT-PCR reactions were performed using RT2 SYBR Green qPCR Mastermix reagent (Qiagen) on an Applied QuantStudio 6 Flex qPCR system (Life Technologies, Grand Island, NY, USA). No template control samples served as negative controls. Differential gene expression was calculated using the 2^−ΔΔCt^ method (genes of interest normalized to β-actin). The data are shown as fold changes in comparison to control groups.

### 2.5. Statistics

All data are presented as the means ± SEM and were compared using a one-way ANOVA or a two-tailed *t*-test. Statistical analyses were conducted using GraphPad Prism 8 software (GraphPad Software Inc., La Jolla, CA, USA). For multiple comparisons among treatment groups, a one-way ANOVA was utilized, followed by Tukey’s multiple comparison tests. Differences among the groups were considered statistically significant at *p*-values below < 0.05.

## 3. Results

### 3.1. Effect of Blast Exposure on Plasma and CSF Melatonin Levels

Several studies indicated that the plasma melatonin concentrations in rats follow diurnal rhythmicity with relatively lower levels during daytime, which increase several-fold upon exposure to dark during nighttime [28,29]. We confirmed these findings by determining the plasma concentration of melatonin in nonblasted rats during daytime and at night. 

**Figure 1:**
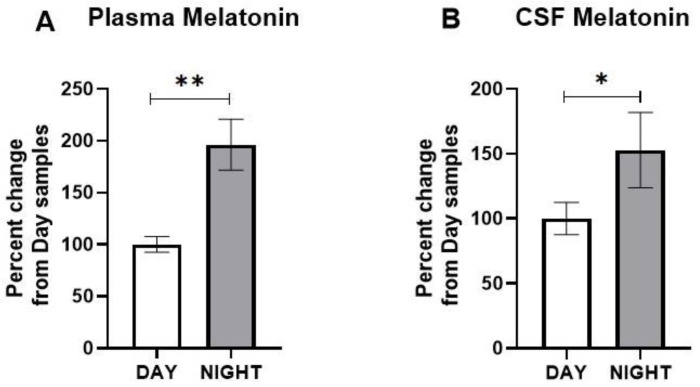
Melatonin concentrations during the day and at night in the (**A**) plasma and (**B**) cerebrospinal fluid (CSF) of nonblasted rats. The results are expressed as means ± SEM (*n* = 6), and the melatonin values of samples collected during daytime were compared to those collected at night (* *p* < 0.05 and ** *p* < 0.01).

The plasma concentration showed a statistically significant increase at night in comparison to the daytime samples (t = 3.727; *p* = 0.0098) (Figure 1A). Similarly, we found an increase in the CSF melatonin concentration at night in comparison to the daytime samples (t = 3.338; *p* = 0.0157) (Figure 1B).

**Figure 2:**
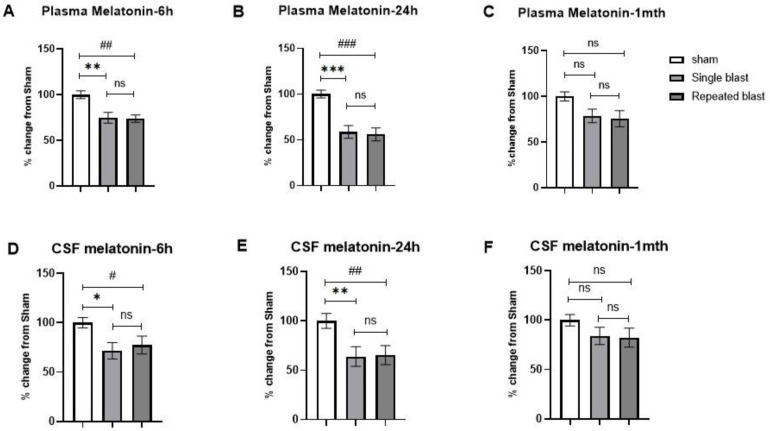
Effect of single and repeated blast exposure on plasma and CSF melatonin concentrations measured at night in SD rats. Plasma melatonin concentrations at (**A**) 6 h, (**B**) 24 h, and (**C**) 1 month after blast injury. CSF melatonin concentrations at (**D**) 6 h, (**E**) 24 h, and (**F**) 1 month after blast injury. Results are expressed as means ± SEM (n = 4–6); *** *p* < 0.001, ** *p* < 0.01, and * *p* < 0.05 for sham vs. single blast group; ### *p* < 0.001, ## *p* < 0.01, and # *p* < 0.05 for sham vs. repeated blast group; ns—not significant.

Based on these results, we next investigated the effects of single and repeated blasts on the plasma and CSF melatonin concentrations in the rats. For samples collected at night, we observed a statistically significant decrease in the plasma concentrations of melatonin at 6 h (F (2, 15) = 8.864; *p* = 0.003) (Figure 2A) and at 24 h (F (2, 15) = 15.68; *p* = 0.0002) (Figure 2B) and a nonsignificant decrease at 1 month (F (2, 9) = 1.909; *p* = 0.2036) (Figure 2C). Similar effects were noted with CSF at 6 h (F (2, 15) = 3.771; *p* = 0.0471) (Figure 2D) and at 24 h (F (2, 15) = 5.004; *p* = 0.0216) (Figure 2E), with a nonsignificant decrease at 1 month (F (2, 15) = 1.396; *p* = 0.278) (Figure 2F). At all three time points, there were no statistically significant differences between the sham group and the blast exposure groups for samples (plasma and CSF) collected during daytime (data not shown).

### 3.2. Effect of Blast Exposure on Expression of Melatonin-Synthesizing Enzymes

The findings that blast-exposed rats exhibited decreases in plasma and CSF melatonin levels prompted us to examine whether the gene expression of enzymes responsible for pineal melatonin synthesis is impaired in blast-exposed rats in comparison to sham rats. 

**Figure 3:**
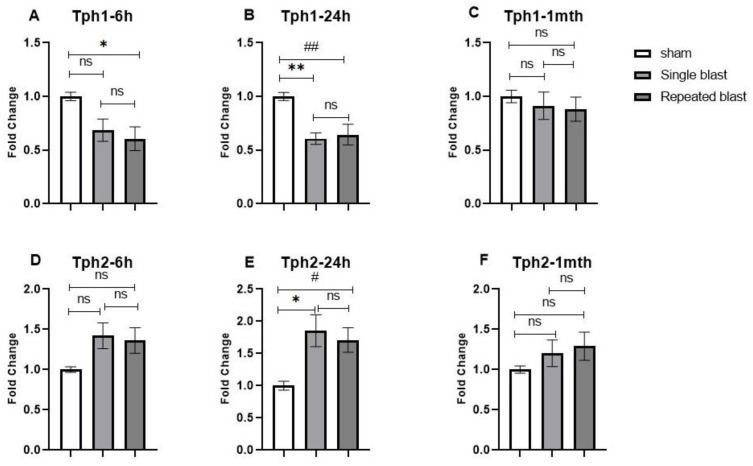
Effect of single and repeated blast exposure on Tph1 and Tph2 mRNA levels at night in the pineal glands of SD rats. Tph1 mRNA concentrations at (**A**) 6 h, (**B**) 24 h, and (**C**) 1 month after blast injury. Tph2 mRNA concentrations at (**D**) 6 h, (**E**) 24 h, and (**F**) 1 month after blast injury. Results are expressed as means ± SEM (n = 6); ** *p* < 0.01 and * *p* < 0.05 for sham vs. single blast group; ## *p* < 0.01 and # *p* < 0.05 for sham vs. repeated blast group; ns—not significant.

The first step in the melatonin synthesis pathway is the conversion of the amino acid l-tryptophan into serotonin, a reaction catalyzed by the activity of the enzyme tryptophan hydroxylase (TPH). Two Tph genes have been described, Tph1 and Tph2. Tph1 is predominantly located in the peripheral organs but is highly expressed in the pineal gland, whereas Tph2 is abundantly present in other regions of the brain [30]. A 2–3-fold increase in nocturnal TPH activity in the pineal gland mediated by sympathetic β-adrenergic stimulation has been reported [31,32,33]. The Tph1 mRNA expression showed a statistically significant decrease in single and repeated blast-exposed rats at 6 h (F (2, 15) = 5.288; *p* = 0.0183) (Figure 3A) and 24 h (F (2, 15) = 10.33; *p* = 0.0015) (Figure 3B), with values reaching near normal levels at 1 month postblast (F (2, 15) = 0.3505; *p* = 0.7100) (Figure 3C). Interestingly, we saw a statistically significant upregulation in Tph2 mRNA levels at 24 h (F (2, 15) = 6.080; *p* = 0.0116) (Figure 3D). Although there was a modest upregulation in Tph2 mRNA expression, it was not statistically significant at 6 h (F (2, 15) = 2.973; *p* = 0.088) (Figure 3E) or 1 month postblast in either group in comparison to the sham rats (F (2, 15) = 1.102; *p* = 0.3576) (Figure 3F).

**Figure 4:**
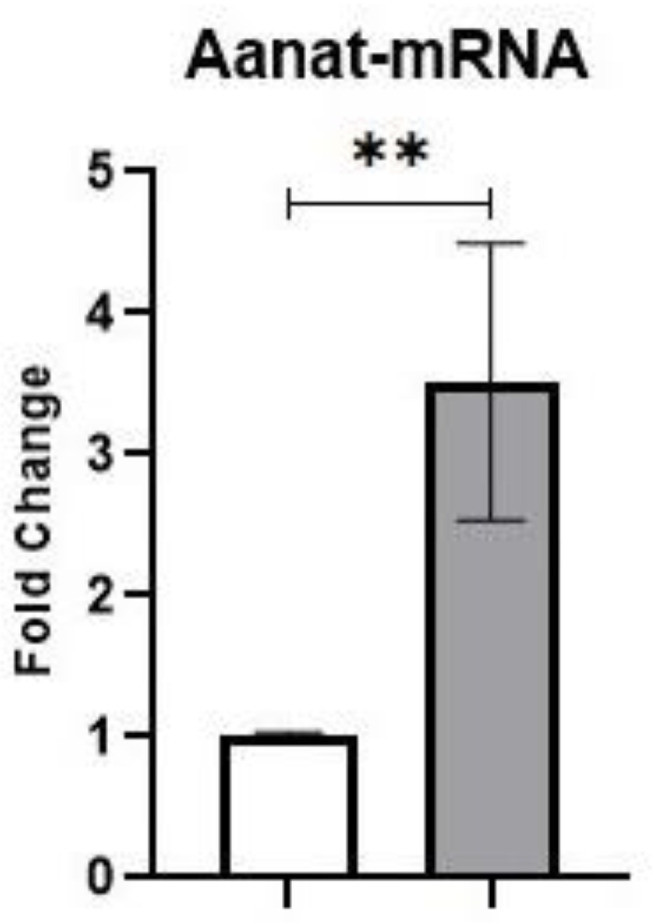
Aanat mRNA concentrations during day and night in the pineal glands of nonblasted rats. Results are expressed as means ± SEM (n = 6); ** *p* < 0.01 in comparison with samples collected during daytime.

The serotonin formed by the action of TPH1 and TPH2 undergoes two additional chemical modifications. The first step is catalyzed by the AANAT enzyme to yield *N*-acetyl serotonin, followed by the transfer of a methyl group from S-adenosyl methionine to the 5-hydroxy group of *N*-acetyl serotonin by ASMT. The transcriptional changes and activity of AANAT, along with melatonin synthesis and secretion, follow a circadian rhythm that is attributed to changes in the light–dark cycle. Since AANAT determines the circadian melatonin rhythm, we examined the diurnal changes in Aanat mRNA expression levels in the pineal gland in nonblasted rats. A statistically significant increase in Aanat-mRNA expression was noted at night in comparison to daytime (t = 2.560; *p* = 0.0284) (Figure 4).

**Figure 5:**
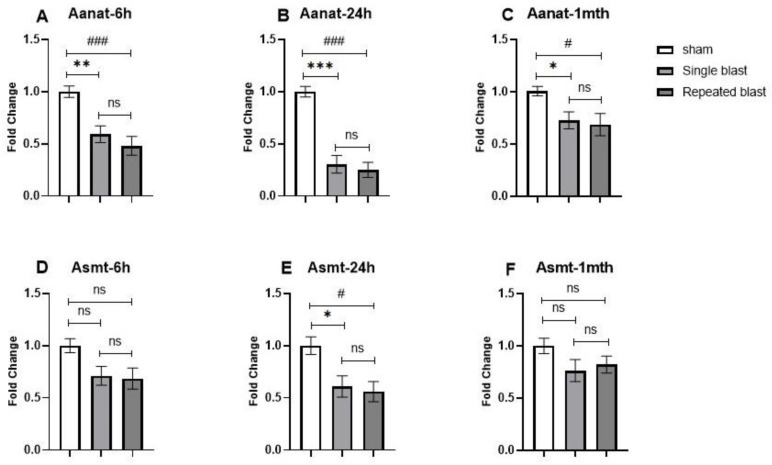
Effects of single and repeated blast exposure on Aanat and Asmt mRNA levels at night in the pineal glands of rats. Aanat mRNA concentrations at (**A**) 6 h, (**B**) 24 h, and (**C**) 1 month after blast injury. Asmt mRNA concentrations at (**D**) 6 h, (**E**) 24 h, and (**F**) 1 month after blast injury. Results are expressed as means ± SEM (n = 6); *** *p* < 0.001, ** *p* < 0.01, and * *p* < 0.05 for sham vs. single blast group; ### *p* < 0.001, and # *p* < 0.05 for sham vs. repeated blast group; ns—not significant.

Next, we investigated the effect of blast exposure on circadian Aanat expression and observed a significant decrease in Aanat mRNA expression in the pineal glands of blast-exposed rats at night. The levels of Aanat mRNA after single and repeated blast exposure showed significant decreases at 6 h (F (2, 15) = 12.6; *p* = 0.0006) (Figure 5A), 24 h (F (2, 15) = 36.07; *p* < 0.0001) (Figure 5B), and 1 month (F (2, 15) = 6.013; *p* = 0.0121) (Figure 5C) in comparison to nonblasted rats. The extent of the decreases in Aanat mRNA levels were comparatively less at 1 month postblast in comparison to 6 h and 24 h. The Asmt mRNA expression showed a statistically significant decrease in single and repeated blast-exposed rats at 24 h (F (2, 15) = 6.489; *p* = 0.0093) (Figure 5E) after blast injury. There were no statistically significant differences at 6 h (F (2, 15) = 3.742; *p* = 0.0480) (Figure 5D) or 1 month (F (2, 15) = 1.949; *p* = 0.1768) (Figure 5F) postblast in comparison to sham rats.

### 3.3. Effect of Blast Exposure on Expression of Melatonin Receptors

**Figure 6:**
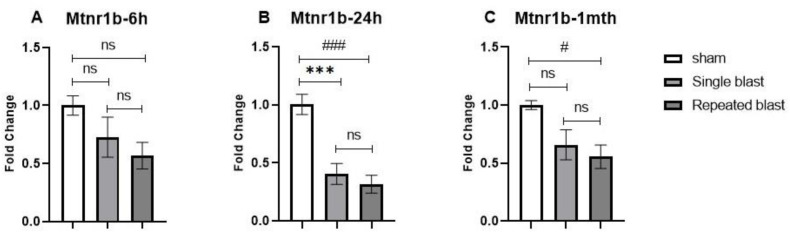
Effect of single and repeated blast exposure on Mtnr1b mRNA levels at night in the pineal glands of SD rats at (**A**) 6 h, (**B**) 24 h, and (**C**) 1 month after blast injury. Results are expressed as means ± SEM (n = 6); *** *p* < 0.001, for sham vs. single blast group; ### *p* < 0.001, and # *p* < 0.05 for sham vs. repeated blast group; ns—not significant.

Since we saw a decrease in the plasma levels of melatonin and changes in the gene expression of various enzymes regulating melatonin synthesis, we investigated the effect of single and repeated blast exposure on the mRNA expression of melatonin receptors in the pineal gland. In humans and mammals, two types of melatonin receptors are identified, namely MTNR1A (MT1 or Mel1a) and MTNR1B (MT2 or Mel1b), each serving different functions [34]. The expression of melatonin receptors in the pineal glands of rats has been less studied. In our study, the levels of mRNA for the Mtnr1a (MT1) receptor subtype were very low and had relatively high CT values (>35). The Mtnr1b mRNA expression was decreased at all time points in both the single and repeated blast groups in comparison to the sham group. At 6 h postblast, although we saw a reduction in Mtnr1b mRNA expression, it was not statistically significant (F (2, 15) = 2.873; *p* = 0.0878) (Figure 6A). At 24 h after blast injury, the Mtnr1b mRNA expression showed a statistically significant decrease in single and repeated blast-exposed rats (F (2, 15) = 19.33; *p* < 0.0001) (Figure 6B). At 1 month postblast, we noted a statistically significant decrease in Mtnr1b mRNA expression in the repeated blast group when compared to the sham group (F (2, 15) = 5.690; *p* = 0.0145) (Figure 6C).

## 4. Discussion

To date, the effect of blast exposure on melatonin synthesis and release in the rodent pineal gland has not been investigated. The results from this study are the first to report that blast-exposed rats exhibit (1) disrupted circadian rhythms associated with decreased mRNA levels of melatonin-synthesizing enzymes and decreased melatonin levels in the CSF and plasma at night and (2) a reduction in mRNA levels of melatonin receptors (Mtnr1b) in the pineal gland at night after blast exposure.

Melatonin synthesis predominantly occurs in the pineal gland and plays a crucial role in regulating several physiological functions, including the maintenance of the circadian rhythm [35]. Previous studies have shown that melatonin synthesis and secretion are lower during the daytime and reach peak values at night in humans and rodents [36,37,38]. Similarly, in the present study, melatonin concentrations in the plasma and CSF of nonblasted rats were low during the daytime (1000 h) and increased in samples collected at night (2200 h). The circadian regulation of sleep and wakefulness is determined by changes in endogenous melatonin levels [39]. As discussed earlier, the circadian rhythm of melatonin production is impaired following TBI, resulting in sleep disturbances, including insomnia and altered sleep–wake cycles [40,41]. In the long term, sleep disturbance is associated with fatigue, depression, anxiety, and cognitive dysfunction, likely due to lower evening melatonin levels [42]. Deficiencies in sleep may also impair recovery from brain injury by increasing the catabolic rate, decreasing cellular and humoral immunity, and impairing cell division, thus compromising the quality of life [43]. In our study, melatonin concentrations in the plasma and CSF in samples collected during the night were decreased to a greater extent in blast-exposed rats than in sham rats, suggesting that blast-exposed rats have a diminished circadian rhythm of melatonin secretion. This is in line with studies conducted in humans, where TBI patients exhibited decreased melatonin production at night, revealing a disruption of circadian rhythmicity [23,25,44].

To gain insight into the molecular mechanisms underlying the reduced nighttime melatonin levels, we investigated the changes in the expression levels of various enzymes (TPH1, TPH2, AANAT, and ASMT) involved in the melatonin synthesis of blast-exposed rats at nighttime (2200 h). These enzymes regulate the synthesis of pineal melatonin in a circadian fashion. The synthesis of melatonin in the pineal gland involves the sequential conversion of L-tryptophan into hydroxytryptophan by the action of the TPH enzyme, followed by the formation of serotonin by the decarboxylase enzyme [15]. Tryptophan hydroxylase (TPH1 and TPH2) is the rate-limiting enzyme in the synthesis of serotonin and plays an important role in the nocturnal synthesis of melatonin in the pineal gland. A 2–3-fold increase in the activity of pineal tryptophan hydroxylase at night to induce melatonin synthesis has been reported [31,32,33]. Two important enzymes, AANAT and ASMT, are expressed at very high levels in the pineal gland and catalyze the conversion of serotonin to melatonin [45]. The high levels of melatonin at night are due to increased activity of AANAT, with few cyclical changes noted in ASMT activity over 24 h in the pineal gland [46]. A modest decrease in Aanat gene expression in the rat frontal cortex of blast-exposed animals has been reported [47]. However, no studies have investigated the effect of blast injury on pineal gland Aanat and Asmt levels. The enzyme AANAT is considered to be the key regulator of melatonin synthesis; the level of the Aanat gene is not detectable during the day, and it increases approximately 100-fold during the night [48]. Various insults to the brain, including TBIs, have been strongly linked to a decrease in circulating melatonin levels at night [23,25]. Our results showing decreased mRNA levels of both Aanat and Asmt in the nighttime, but not in the daytime, after blast exposure suggest that blast exposure affects the circadian melatonin rhythm by decreasing the pineal gland synthesis of melatonin associated with the sleep–wake cycle. 

The enzyme TPH has two isoforms: TPH1 and TPH2. The TPH1 isoform is predominantly located in the peripheral organs but is also highly expressed in the pineal gland, whereas TPH2 is abundantly expressed in other regions of the brain [49]. Furthermore, in normal individuals, TPH1 shows an approximately 4-fold nocturnal increase, whereas TPH2 in the pineal gland does not significantly vary across the time of the day, indicating the selectively significant role of TPH1 in the circadian melatonin rhythm [30,50]. In our study, we noted decreased expression of Tph1 and a corresponding increase in the Tph2 expression levels in the blast-exposed rats when compared to nonblasted rats. The effect of blast exposure on pineal gland Tph1 mRNA expression had not been investigated previously; however, a study by Osier et al. showed increased Tph2 expression from 2 h up to 24 h after single blast exposure in the locus coeruleus and dorsal raphe nucleus of SD rats [51]. The increase in Tph2 mRNA levels in the pineal gland after blast exposure could be a compensatory response to the reduced Tph1 levels, which could perform a major function in converting L-tryptophan to hydroxytryptophan. 

Melatonin receptors are highly expressed in different regions of the brain [52], and a limited number of studies have demonstrated the expression of melatonin receptors in the pineal gland. Our novel finding that blast exposure induces a significant reduction in the mRNA level of the melatonin Mtnr1b receptor in the rat pineal gland indicates that the melatoninergic system is a potential therapeutic target in blast-induced sleep disruptions. In a rat model of TBI, reduced melatonin receptors (MTNR1a and MTNR1b) in the frontal cortex and the hippocampus were noted up to 24 h postinjury. The levels of melatonin receptors were also found to be decreased in an animal model of depression [53]. This reduction in melatonin receptors in the pineal gland may affect the efficacy of melatonin treatment in TBI patients, which mandates further investigations. This emerging line of studies indicates the identification of different regions of the brain affected by blast exposure and provides a basis for precision therapies. 

There are certain limitations that need consideration when interpreting the results of this study. The results need to be confirmed in a diurnal animal model to mimic the changes in humans since our results were obtained from rats, which are nocturnal rodents. The rt-PCR mRNA levels only provide limited evidence for the changes noted in the blast-exposed groups. Additional techniques to quantify protein expression, enzyme activity, and any post-translational changes to the studied enzymes also need to be applied and evaluated. The circadian rhythm involves cross-talk among many regions of the brain (primarily the suprachiasmatic nucleus and the raphe nucleus) with the pineal gland. Changes in the mRNA and protein expressions of enzymes and receptors associated with the circadian rhythm in these regions of the brain would provide better insights into the neurobiological alterations involved in circadian rhythm dysregulation after blast exposure.

## 5. Conclusions

The results obtained from our study suggest that blast exposure affects the pineal gland synthesis of melatonin in the nighttime, but not in the daytime, by the differential dysregulation of enzymes involved in its synthetic pathway, leading to decreased melatonin levels in the CSF and plasma. This decreased nighttime synthesis and release of melatonin after blast exposure might be at least partially responsible for the sleep disruptions reported in blast-exposed victims. Further studies are required to investigate the interplay among the pineal gland and the brain regions regulating the circadian melatonin rhythm and to replicate the current findings with diurnal animal models.

## Figures and Tables

**Table 1:** List of primers.

Gene	RefSeq Accession Number	Catalog Number
Tph1	NM_001100634.2	PPR54282B
Tph2	NM_173839.2	PPR48244A
Aanat	NM_012818.2	PPR52708B
Asmt	NM_144759.2	PPR45038B
Mtnr1a	NM_053676.2	PPR53055A
Mtnr1b	NM_001100641.2	PPR52453A
β-Actin	NM_031144.3	PPR06570C

## Data Availability

Not applicable.

## References

[1] Collen J., Orr N., Lettieri C., Carter K., Holley A.B. (2012). Sleep Disturbances among Soldiers with Combat-Related Traumatic Brain Injury. Chest.

[2] Baumann C., Werth E., Stocker R., Ludwig S., Bassetti C.L. (2007). Sleep-wake disturbances 6 months after traumatic brain injury: A prospective study. Brain.

[3] Serin Y., Tek N.A. (2019). Effect of Circadian Rhythm on Metabolic Processes and the Regulation of Energy Balance. Ann. Nutr. Metab..

[4] Sandsmark D.K., Elliott J., Lim M.M. (2017). Sleep-Wake Disturbances After Traumatic Brain Injury: Synthesis of Human and Animal Studies. Sleep.

[5] Viola-Saltzman M., Musleh C. (2016). Traumatic brain injury-induced sleep disorders. Neuropsychiatr. Dis. Treat..

[6] Zisapel N. (2018). New perspectives on the role of melatonin in human sleep, circadian rhythms and their regulation. J. Cereb. Blood Flow Metab..

[7] Vasey C., McBride J., Penta K. (2021). Circadian Rhythm Dysregulation and Restoration: The Role of Melatonin. Nutrients.

[8] Liu C., Weaver D.R., Strogatz S.H., Reppert S.M. (1997). Cellular Construction of a Circadian Clock: Period Determination in the Suprachiasmatic Nuclei. Cell.

[9] Darlington T.K., Wager-Smith K., Ceriani M.F., Staknis D., Gekakis N., Steeves T.D.L., Weitz C.J., Takahashi J.S., Kay S.A. (1998). Closing the Circadian Loop: CLOCK-Induced Transcription of Its Own Inhibitors *per* and *tim*. Science.

[10] Ono D., Honma K.-I., Honma S. (2015). Circadian and ultradian rhythms of clock gene expression in the suprachiasmatic nucleus of freely moving mice. Sci. Rep..

[11] Curtis A.M., Bellet M.M., Sassone-Corsi P., O’Neill L.A. (2014). Circadian Clock Proteins and Immunity. Immunity.

[12] Zavala E., Voliotis M., Zerenner T., Tabak J., Walker J.J., Li X.F., Terry J.R., Lightman S., O’Byrne K., Tsaneva-Atanasova K. (2020). Dynamic Hormone Control of Stress and Fertility. Front. Physiol..

[13] Amaral F.G., Castrucci A.M., Cipolla-Neto J., Poletini M.O., Mendez N., Richter H.G., Sellix M.T. (2014). Environmental Control of Biological Rhythms: Effects on Development, Fertility and Metabolism. J. Neuroendocr..

[14] Sciarra F., Franceschini E., Campolo F., Gianfrilli D., Pallotti F., Paoli D., Isidori A.M., Venneri M.A. (2020). Disruption of Circadian Rhythms: A Crucial Factor in the Etiology of Infertility. Int. J. Mol. Sci..

[15] Xie X., Ding D., Bai D., Zhu Y., Sun W., Sun Y., Zhang D. (2022). Melatonin biosynthesis pathways in nature and its production in engineered microorganisms. Synth. Syst. Biotechnol..

[16] Borjigin J., Wang M.M., Snyder S.H. (1995). Diurnal variation in mRNA encoding serotonin N-acetyltransferase in pineal gland. Nature.

[17] Roseboom P.H., Coon S.L., Baler R., McCune S.K., Weller J.L., Klein D.C. (1996). Melatonin synthesis: Analysis of the more than 150-fold nocturnal increase in serotonin N-acetyltransferase messenger ribonucleic acid in the rat pineal gland. Endocrinology.

[18] Tosini G., Chaurasia S.S., Michael Iuvone P. (2006). Regulation of arylalkylamine N-acetyltransferase (AANAT) in the retina. Chronobiol. Int..

[19] Liu T., Borjigin J. (2005). N-acetyltransferase is not the rate-limiting enzyme of melatonin synthesis at night. J. Pineal Res..

[20] MacGregor A.J., Dougherty A.L., Galarneau M.R. (2011). Injury-Specific Correlates of Combat-Related Traumatic Brain Injury in Operation Iraqi Freedom. J. Head Trauma Rehabil..

[21] Eskridge S., Macera C.A., Galarneau M.R., Holbrook T.L., Woodruff S.I., MacGregor A.J., Morton D.J., Shaffer R.A. (2012). Injuries from combat explosions in Iraq: Injury type, location, and severity. Injury.

[22] Lucci E.B. (2006). Civilian Preparedness and Counter-terrorism: Conventional Weapons. Surg. Clin. N. Am..

[23] Shekleton J.A., Parcell D.L., Redman J.R., Phipps-Nelson J., Ponsford J.L., Rajaratnam S.M.W. (2010). Sleep disturbance and melatonin levels following traumatic brain injury. Neurology.

[24] Osier N., McGreevy E., Pham L., Puccio A., Ren D., Conley Y.P., Alexander S., Dixon C.E. (2018). Melatonin as a Therapy for Traumatic Brain Injury: A Review of Published Evidence. Int. J. Mol. Sci..

[25] Grima N.A., Ponsford J.L., Hilaire M.A.S., Mansfield D., Rajaratnam S.M. (2016). Circadian Melatonin Rhythm Following Traumatic Brain Injury. Neurorehabilit. Neural Repair.

[26] Bekała A., Płotek W., Siwicka-Gieroba D., Sołek-Pastuszka J., Bohatyrewicz R., Biernawska J., Kotfis K., Bielacz M., Jaroszyński A., Dabrowski W. (2022). Melatonin and the Brain–Heart Crosstalk in Neurocritically Ill Patients—From Molecular Action to Clinical Practice. Int. J. Mol. Sci..

[27] Arun P., Wilder D.M., Eken O., Urioste R., Batuure A., Sajja S., Van Albert S., Wang Y., Gist I.D., Long J.B. (2020). Long-Term Effects of Blast Exposure: A Functional Study in Rats Using an Advanced Blast Simulator. J. Neurotrauma.

[28] Ozaki Y., Lynch H.J., Wurtman R.J. (1976). Melatonin in Rat Pineal, Plasma, and Urine: 24-Hour Rhythmicity and Effect of Chlorpromazine. Endocrinology.

[29] Dauchy R.T., Wren M.A., Dauchy E.M., Hoffman A.e., Hanifin J.P., Warfield B., Jablonski M.R., Brainard G.C., Hill S.M., Mao L. (2015). The influence of red light exposure at night on circadian metabolism and physiology in Sprague-Dawley rats. J. Am. Assoc. Lab. Anim. Sci..

[30] Walther D.J., Peter J.U., Bashammakh S., Hörtnagl H., Voits M., Fink H., Bader M. (2003). Synthesis of Serotonin by a Second Tryptophan Hydroxylase Isoform. Science.

[31] Shibuya H., Toru M., Watanabe S. (1977). A circadian rhythm of tryptophan hydroxylase in rat pineals. Brain Res..

[32] Sitaram B.R., Lees G.J. (1978). Diurnal rhythm and turnover of tryptophan hydroxylase in the pineal gland of the rat. J. Neurochem..

[33] Sugden D., Grady R., Mefford I.N. (1989). Measurement of Tryptophan Hydroxylase Activity in Rat Pineal Glands and Pinealocytes Using an HPLC Assay With Electrochemical Detection. J. Pineal Res..

[34] Emet M., Ozcan H., Ozel L., Yayla M., Halici Z., Hacimuftuoglu A. (2016). A Review of Melatonin, Its Receptors and Drugs. Eurasian J. Med..

[35] Pieri C., Marra M., Moroni F., Recchioni R., Marcheselli F. (1994). Melatonin: A peroxyl radical scavenger more effective than vitamin E. Life Sci..

[36] Lewy A.J., Wehr T.A., Goodwin F.K., Newsome D.A., Markey S.P. (1980). Light Suppresses Melatonin Secretion in Humans. Science.

[37] Aoki H., Yamada N., Ozeki Y., Yamane H., Kato N. (1998). Minimum light intensity required to suppress nocturnal melatonin concentration in human saliva. Neurosci. Lett..

[38] Badness T.J., Powers J.B., Hastings M.H., Bittman E.L., Goldman B.D. (1993). The timed infusion paradigm for melatonin delivery: What has it taught us about the melatonin signal, its reception, and the photoperiodic control of seasonal responses?. J. Pineal Res..

[39] Rajaratnam S.M., Cohen D.A., Rogers N.L. (2009). Melatonin and Melatonin Analogues. Sleep Med. Clin..

[40] Cohen M., Oksenberg A., Snir D., Stern M.J., Groswasser Z. (1992). Temporally related changes of sleep complaints in traumatic brain injured patients. J. Neurol. Neurosurg. Psychiatry.

[41] Ouellet M.C., Beaulieu-Bonneau S., Morin C.M. (2006). Insomnia in patients with traumatic brain injury: Frequency, characteristics, and risk factors. J. Head Trauma Rehabil..

[42] Ponsford J.L., Ziino C., Parcell D.L., Shekleton J.A., Roper M., Redman J.R., Phipps-Nelson J., Rajaratnam S. (2012). Fatigue and Sleep Disturbance Following Traumatic Brain Injury—Their Nature, Causes, and Potential Treatments. J. Head Trauma Rehabil..

[43] Shilo L., Dagan Y., Smorjik Y., Weinberg U., Dolev S., Komptel B., Shenkman L. (2000). Effect of melatonin on sleep quality of copd intensive care patients: A pilot study. Chronobiol. Int..

[44] Shilo L., Dagan Y., Smorjik Y., Weinberg U., Dolev S., Komptel B., Balaum H., Shenkman L. (1999). Patients in the Intensive Care Unit Suffer from Severe Lack of Sleep Associated with Loss of Normal Melatonin Secretion Pattern. Am. J. Med. Sci..

[45] Maronde E., Stehle J.H. (2007). The mammalian pineal gland: Known facts, unknown facets. Trends Endocrinol. Metab..

[46] Klein D.C. (2007). Arylalkylamine N-acetyltransferase:“The Timezyme”. J. Biol. Chem..

[47] Haghighi F., Ge Y., Chen S., Xin Y., Umali M.U., De Gasperi R., Sosa M.A.G., Ahlers S.T., Elder G.A. (2015). Neuronal DNA Methylation Profiling of Blast-Related Traumatic Brain Injury. J. Neurotrauma.

[48] Oliveira P.F., Sousa M., Monteiro M.P., Silva B., Alves M.G., Skinner M.K. (2018). Pineal Gland and Melatonin Biosynthesis. Encyclopedia of Reproduction.

[49] Walther D.J., Bader M. (2003). A unique central tryptophan hydroxylase isoform. Biochem. Pharmacol..

[50] Sugden D. (2003). Comparison of circadian expression of tryptophan hydroxylase isoform mRNAs in the rat pineal gland using real-time PCR. J. Neurochem..

[51] Osier N.D., Pham L., Pugh B.J., Puccio A., Ren D., Conley Y.P., Alexander S., Dixon C.E. (2017). Brain injury results in lower levels of melatonin receptors subtypes MT1 and MT2. Neurosci. Lett..

[52] Ishii H., Tanaka N., Kobayashi M., Kato M., Sakuma Y. (2009). Gene structures, biochemical characterization and distribution of rat melatonin receptors. J. Physiol. Sci..

[53] Wang S., Tian Y., Song L., Lim G., Tan Y., You Z., Chen L., Mao J. (2012). Exacerbated mechanical hyperalgesia in rats with genetically predisposed depressive behavior: Role of melatonin and NMDA receptors. Pain.

